# Systems Biology: The Role of Engineering in the Reverse Engineering of Biological Signaling

**DOI:** 10.3390/cells2020393

**Published:** 2013-05-31

**Authors:** Pablo A. Iglesias

**Affiliations:** Department of Electrical and Computer Engineering, The Johns Hopkins University, 3400 N. Charles Street, Baltimore, MD 21218, USA; E-Mail: pi@jhu.edu; Tel.: +1-410-516-6026; Fax: +1-410-516-5566

**Keywords:** control engineering, information theory, signal processing, statistical inference, homeostasis

## Abstract

One of the principle tasks of systems biology has been the reverse engineering of signaling networks. Because of the striking similarities to engineering systems, a number of analysis and design tools from engineering disciplines have been used in this process. This review looks at several examples including the analysis of homeostasis using control theory, the attenuation of noise using signal processing, statistical inference and the use of information theory to understand both binary decision systems and the response of eukaryotic chemotactic cells.

## 1. Introduction

Though no two researchers are likely to agree on a definition of systems biology, there is no doubt that an important aspect is the way that it integrates research from an array of disciplines by combining novel experimental and computational tools. The various engineering disciplines have contributed many tools that facilitate experimentation, computation and data processing. Equally important is the role that our understanding of the engineering design process can have in deciphering biological systems. Issues such as modularity, robustness, optimality, tradeoffs, and physical constraints are dealt with every day in engineering design, and a large number of theoretical tools have been developed to facilitate this process. In studying biology, it is important to understand that these tradeoffs and constraints also arise and hence must be dealt with [1,2].

This review highlights how some of this understanding has played a role in the reverse engineering of biological systems. The emphasis is on a number of theoretical tools from research areas in engineering that in some sense deal with abstract representations of signals and systems and hence are readily amenable for use in system biology, particularly in the study of signal transduction pathways. An example is the set of design and analysis tools developed by control engineers. Though this field grew out of different application areas in chemical, electrical and aerospace engineering [3], the tools used by control engineers are usually not specific to any particular application, but instead focus on signals that need to be controlled and mathematical descriptions, rather than hardware implementation, of systems that perform that regulation. Thus, control engineering tools have found applications in the systems biology research [4–9]. Similarly, in signal processing and information theory, the emphasis is placed on the statistical properties of signals and how to alter these to achieve the desired objectives while emphasizing constraints that are placed by noise.

The rest of this review is divided into four parts. The first three look at different engineering disciplines, though it is important to stress that even within engineering, the divisions between these areas is not always so clear-cut. Finally, some concluding remarks are presented.

## 2. Homeostasis and Control Engineering

Since the work of Claude Bernard in the 19th century, the process by which an organism's internal state is kept constant—*le milieu intérieur*—has long fascinated biologists [10]. In the 20th century, Walter Cannon termed this process *homeostasis* and argued that diseases arose because of a homeostatic imbalance [11]. This imbalance could be due to an inability for the organism to cope with changing environments, either because of a loss of function of its regulatory mechanisms, or because the level of the stimulus is too large for these mechanisms to cope. Cannon proposed six principles that allowed a system to achieve homeostasis. One of these:

If the state remains steady, there is an automatic arrangement whereby any tendency toward change is effectively met by increased action of the factor or factors which resist the change. (Cited in [10].)

clearly expresses the need for negative feedback. The necessity of Cannon's principles, although a result of intuition based on his understanding of human physiology, can be stated in a mathematically formal way, as we will see below.

### 2.1. Perfect Adaptation and the Internal Model Principle

**Figure 1.**
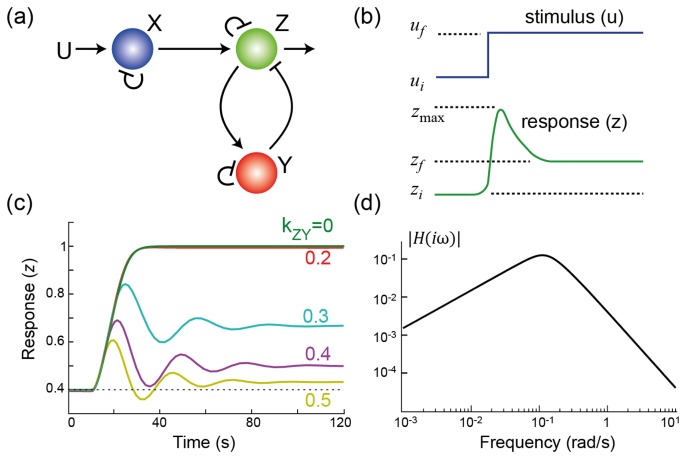
**Adaptation through negative feedback. (a)** Three node network involving negative feedback regulation through node *Y* ; **(b)** This cartoon shows a partially adapting system. As the level of the stimulus increases from its initial (*u_i_*) to final (*u_f_*) level, the response shows a biphasic response. It increases from its initial value (*z_i_*), reaches a peak (*z*_max_) before settling to a final steady-state (*z_f_*). In this case, the system is partially adapting. It is said to be perfectly adapting if *z_i_* = *z_f_*; **(c)** Simulation results of the system in panel (**a**) for varying strength of the feedback loop; **(d)** Frequency response of the system. The slope seen at low frequencies is indicative of integral control. At high frequencies, this drop provides filtering of high frequency components.

To illustrate how feedback arises in the context of a homeostatic response, we use a simple three-node network where an external stimulus (input; denoted *U*) gives rise to a response (output; denoted *Z*) by activating a signaling cascade [12]; see Figure 1(a). Each node in the network represents a signaling element that exists in two states: active and inactive, and the conversion between the two is based on enzymatic reactions, represented by Michaelis–Menten kinetics. Positive interactions increase the rate of the enzymatic reaction activating the product; negative interactions increase the rate of inactivation. The network includes a negative feedback loop achieved through node *Y*. The strength of this interaction can be changed by altering the parameter *k_ZY_*.

Based on these assumptions, the following set of differential equations describes the system, where *x*, *y*, *z* and *u* denote suitably normalized concentrations of the active state of the various components

$$\begin{array}{ll} \frac{\text{d}x(t)}{\text{d}t} & =u(t)k_{UX}\frac{1-x(t)}{1-x(t)+k_{X}}-k_{-X}\frac{x(t)}{x(t)+k_{X}^{\prime }}\\ \frac{\text{d}y(t)}{\text{d}t} & =z(t)k_{ZY}\frac{1-y(t)}{1-y(t)+k_{Y}}-k_{-Y}\frac{y(t)}{y(t)+k_{Y}^{\prime }}\\ \frac{\text{d}z(t)}{\text{d}t} & =x(t)k_{XZ}\frac{1-z(t)}{1-z(t)+k_{Z}}-y(t)k_{-Z}\frac{z(t)}{z(t)+k_{Z}^{\prime }} \end{array}$$

The specific parameter values are not important, but all coefficients are assumed to be positive.

Suppose that we are interested in making the system insensitive to changes in the stimulus concentration. Because this is a dynamical system, we need to be more precise as to the class of inputs to which we would like to be insensitive, and we need a means for measuring sensitivity. For the former, it is customary to consider changes in the set point of the system—that is, we assume that the stimulus is at a steady-state value, *u_i_*, and that at a given time, *t =* 0, it is changed to a new value *u_f_* > *u_i_*. This *step* change in concentration gives rise to a transient change in the response from its pre-stimulus value (*z_i_*), before settling to a new steady-state (*z_f_*); see Figure 1(b). The *precision*, or steady-state sensitivity, is the fractional change in the concentration of the response, relative to the fractional change in the stimulus: *P* = ((*u_f_* − *u_i_*)/*u_i_*)/((*z_f_* − *z_i_*)/*z_i_*) [12]. In general, this is a nonlinear function, so that the initial stimulus level makes a difference. The analysis can be simplified if we assume that the system is initially at rest; that is, *u_i_* = 0, from which *x_i_* = *y_i_* = *z* = 0 follows. In this case, the definition above is not suitable; instead, we let *P* = *u_f_*/*z_f_*. With either definition, if the system output returns to the initial value, that is, it is completely insensitive to the external stimulus, then the precision is infinite and the system is said to achieve *perfect adaptation*.

Figure 1(c) illustrates the effect that varying the strength of the negative feedback has on the system. Note that, for a given feedback strength, the response increases after the change in the stimulus level. However, as the strength of the negative feedback is increased, the sensitivity decreases. To make the system adapt perfectly requires an infinitely large negative feedback gain. This is obviously not possible in general, but can be achieved in certain cases. If the negative feedback pathway is operating at saturation (1 − *y* ≫ *k_Y_* and $y\gg k_{Y}^{\prime }$) then the differential equation for the feedback is approximately

(1) $$\begin{array}{ll} \frac{dy(t)}{dt} & \approx z(t)k_{ZY}-k_{-Y}\\ & =-k_{ZY}(z*-z(t)) \end{array}$$

where z* = *k*_−_*_Y_*/*k_ZY_* is the steady-state set-point, which is independent of the stimulus level. Evaluating this equation at the equilibrium (where the derivative is zero) shows that the output returns to the set-point that is independent of the level of stimulus. Integrating Equation 1 yields

$$y(t)\approx -k_{ZY}\int_{0}^{t}e(\tau )\;\text{d}\tau$$

where *e*(*t*) = z* − *z*(*t*) is the deviation of the output from its set point. It is clear from this expression that the feedback connection is integrating changes from the set-point. In engineering, this form of feedback is referred to as *integral control*, and it is well known that for a system to be completely insensitive to external step changes in the set point, like those considered here, the system must have integral control feedback [13]. Moreover, this is a special case of a more general set of results known as the *Internal Model Principle* (IMP), which states that for a system to be robustly insensitive to an external change in its environment, it must have both a negative feedback connection (echoing Cannon's postulate) and an internal dynamical system that can recreate the class of disturbances [14,15]. Specifically, integrators recreate step disturbances, but other classes of stimuli are possible and require other forms of negative feedback.

To illustrate how integral feedback is providing infinite gain, it is useful to write the integrator in terms of a Fourier transform. In this case,

(2) $$Y(i\omega )=-\frac{k_{ZY}}{i\omega }E(i\omega )$$

The ratio between the Fourier transforms of the error *e*(*t*) and feedback signal *y*(*t*) is known as the *transfer function* and, in this case, equals −*k_ZY_*/(*iω*). In particular, we see that at steady-state, which corresponds to frequency *ω* = 0, there is an infinite gain between the deviation from steady-state and the control signal. Thus, though the controller gain is not infinite for all frequencies, it is at the particular frequency where the desired adaptation precision is to be measured (*ω* = 0).

The frequency domain analysis presented above forms one of the strongest tools of control engineering and signal processing. It does require that the particular system be linear, or at least linearized so that only small deviations from the steady-state are considered. In linearization, a signal (e.g., *x*(*t*)) is considered as the sum of two parts: the constant steady-state (x*) and deviations from this steady-state (*δx*(*t*) = *x*(*t*) − x*). If we perform this linearization on the system above and write the corresponding signals in the frequency domain using the Fourier transform, we obtain the closed-loop transfer function

$$H(i\omega )=\frac{\Delta Z(i\omega )}{\Delta U(i\omega )}=\frac{(i\omega )b}{\left((i\omega )+a_{0})({\left(i\omega \right)}^{2}+a_{1}(i\omega )+a_{2}\right)}$$

which describes the effect of a sinusoidal deviation from steady-state on the output; see Figure 1(d). The coefficients in the denominator are all positive (shown in the Appendix), guaranteeing that the system is stable. It is worth noting that the term (*iω*) that appeared in the denominator when describing the controller (Equation 2) is now in the numerator of the closed-loop system. This term says that the gain at steady-state is zero—which is equivalent to saying that the system is attenuating constant signals; in other words, the system is performing temporal differentiation. The IMP states that integral feedback is *required* for perfect adaptation to step changes in the environmental stimuli. Thus, we can expect to see integral feedback in action in biological systems that exhibit perfect adaptation, and this has been the case. The first, and best known, was in the study of the signaling system controlling chemotaxis in *E. coli*. These cells move through the action of a number of flagella. When rotating in a counterclockwise fashion, the flagella form a bundle and propel the cell forward in a *run*. If the rotation is clockwise, the flagella unbundle, and the cell comes to a sudden stop. In this state, the cell undergoes a *tumble*, a random reorientation in space. Through a series of runs and tumbles, the cell performs a random walk through space. In response to chemoattractants, *E. coli* modulates the time between tumbles, lengthening it if moving up a gradient, and shortening when moving away from chemoattractants. This biases the direction of the random walk so that, on average, cells migrate up a chemoattractant gradient. Experimentally, the effect of chemoattractant on the rotational bias can be observed in the lab by monitoring the probability that flagella rotate in clockwise fashion before and after stimulation. After the addition of a persistent stimulus, the probability of a clockwise rotation drops for approximately 3–4 seconds before adapting to its pre-stimulus level. Alon *et al.* showed that the chemotactic system displayed perfect adaptation in spite of genetic mutations that greatly changed the expression levels of various components of the signaling pathway [16]. Analysis of the equations that govern adaptation in bacteria [17] demonstrated that this robustness could be attributed to the presence of an integral control feedback element in the loop [18].

A second example is the network regulating the response to osmotic shock in *S. cerevisiae* yeast cells. When faced by changes in turgor pressure, yeast cells respond by activating the High Osmolarity Glycerol (Hog1) pathway, a mitogen-activated protein kinase (MAPK) cascade [19]. This activation, which involves the dual phosphorylation of the MAPK Hog1, can be detected by tracking fluorescently-tagged Hog1. Inactive Hog1 proteins are usually cytoplasmic but, after phosphorylation and activation, translocate to the nucleus [19]. The osmotic-shock system was known to have multiple feedback loops operating at different time scales, but there had previously been no understanding of their specific biological functions. Mettetal *et al.* applied periodically varying stimuli at different frequencies to both wild-type and mutant cells [20] as well as other stimuli like steps and ramps [21]. By measuring the response to these stimuli, they were able to recreate transfer functions experimentally for the various cells strains, which were used to conclude that the osmo-adaptation response is dominated by the feedback path through the kinase Hog1.

**Figure 2.**
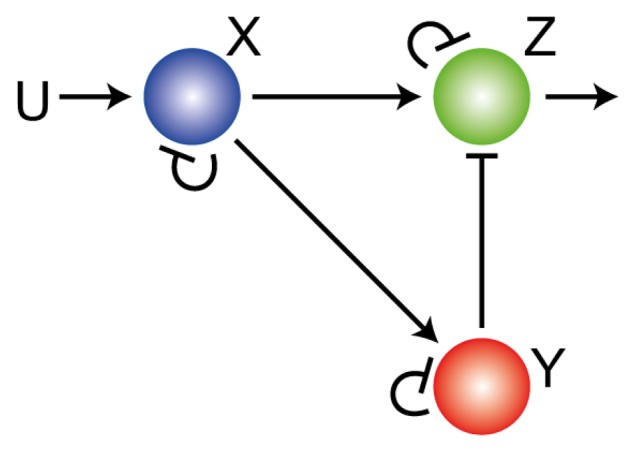
**Adaptation through feedforward regulation.** Three node network showing an incoherent feedforward network that can provide perfect adaptation.

While the IMP states that integral control must be present in order to achieve perfect adaptation to step changes in stimuli, the nature of the integral control may not always be readily apparent. For example, though adaptation in the network of Figure 1 is achieved using saturating negative feedback, there is a second way that adaptation can be achieved in a three node network [12], as illustrated in Figure 2. The external signal *U* increases the production rate of *X*, which increases the production of both *Y* and *Z*. Signal *Y* acts negatively on the response *Z*. Because the external signal regulates the production of *Z* in two complementary ways (positive through *X*; negative through *Y*) the system is said to have an *incoherent feedforward loop* (IFL) [22]. If these regulations are such that forward reactions are saturated (e.g., 1 − *x* ≫ *k_X_*, *etc.*) and the reverse reactions linear (e.g., $k_{X}^{\prime }\gg x$, *etc.*) then:

$$\begin{array}{ll} \frac{\text{d}x(t)}{\text{d}t} & \approx u(t)k_{UX}-k_{-X}x(t)\\ \frac{\text{d}y(t)}{\text{d}t} & \approx x(t)k_{XY}-k_{-Y}y(t)\\ \frac{\text{d}z(t)}{\text{d}t} & \approx x(t)k_{XZ}-k_{-Z}y(t)z(t) \end{array}$$

(In these equations we have incorporated $k_{X}^{\prime }$ into *k*_−_*_X_*, *etc.*) At steady-state, *X* is proportional to the stimulus level (*x_f_* = (*k_UX_*/*k*_−_*_X_*)*u_f_*), *Y* is proportional to *X* (*y_f_* = (*k_XY_*/*k*_−_*_Y_*)*x_f_*) and the response is proportional to the ratio between *X* and *Y*:

$$z_{f}=\frac{k_{XZ}}{k_{-Z}}\frac{x_{f}}{y_{f}}=\frac{k_{XZ}k_{-Y}}{k_{-Z}k_{XY}}$$

and hence is independent of the external stimulus. This mechanism of perfect adaptation was first proposed by Koshland to explain adaptation in *E. coli* [23], but is now more widely accepted as a model for sensory adaptation in eukaryotic cells [24–26]. Note this adaptation is perfect *and* is also extremely robust to parameters [24], though it is not apparent that it includes feedback, much less integral control. However, using a change of coordinates, it can be shown that this system can be recast in terms of an integral feedback [27,28]. This shows that though the IMP holds, its application is not always straightforward.

### 2.2. Fundamental Constraints on Sensitivity Minimization

**Figure 3.**
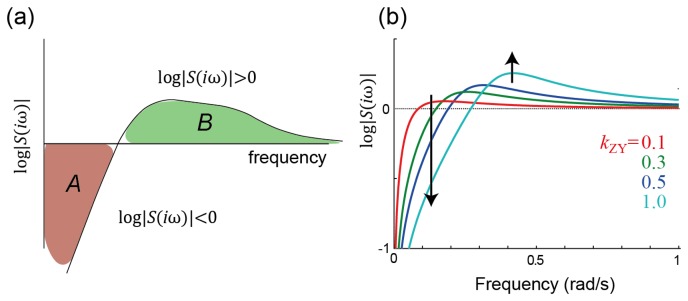
**Frequency-domain constraints on sensitivity. (a)** Waterbed effect and Bode's integral. Bode's integral (Equation 3) states that the total amount of sensitivity attenuation (the area where the gain of the sensitivity function is below one, shown in red and labeled A, must be matched (at least) by the total amount of sensitivity amplification (the green area, marked B). Thus, in the same way that pushing down on one part of a waterbed makes other parts of the bed rise, decreasing the sensitivity in one range of frequencies forces it to increase at other frequencies; **(b)** Illustration of the waterbed effect on the system of Figure 1. Note that, in the time domain, the increase in amplification at higher frequencies shows up as oscillatory behavior; see Figure 1(c).

In the frequency domain, the transfer function *H*(*iω*) exhibits a biphasic response (Figure 1(d)). The (infinitely) small gains at low frequency arise because of the presence of integral control. However, at mid-frequencies, the gain increases, signifying that there are periodic stimuli whose response is actually enhanced. In the time domain, this is manifested as the oscillatory response seen in Figure 1(c). This behavior is a result of a fundamental result from control theory usually referred to as *Bode's Integral Formula*, but more colloquially known as the *waterbed effect*, which places a constraint on the total amount of sensitivity reduction that a system can provide [29,30]; see Figure 3.

To express the sensitivity we consider the transfer function and write this as the product of two functions, *H*(*iω*) = *H*_0_(*iω*)*S*(*iω*) (see Appendix). The first function describes the system without any feedback. The second, referred to as the *sensitivity function*, specifies how feedback reduces the gain of the closed-loop system. In terms of the sensitivity transfer function at various frequencies, Bode's integral states that

(3) $$\int_{0}^{\infty }\log|S(i\omega )|\text{d}\omega \geq 0$$

Though written for systems with transfer functions, this result is considerably more general [31,32]. Moreover, for certain classes of systems, the right-hand side can be made more precise [30]. Nevertheless, as written, this result states that the set of frequencies for which the sensitivity is attenuated (log |*S*(*iω*)| < 0) must be countered by other frequencies for which the signal is amplified (log |*S*(*iω*)| > 0). Moreover, the greater the attenuation is imposed at some frequencies, the larger the amplification must be at others (Figure 3(b)). The regions of frequency over which the sensitivity is attenuated can be said to correspond to regions where the feedback is negative; similarly, sensitivity amplification corresponds to positive feedback. Thus, Bode's integral can be interpreted as implying that in any given system, there must be regions of both negative *and* positive feedback and that these must be balanced. This echoes another of Cannon's dictums on homeostasis:

Factors which may be antagonistic in one region, where they effect a balance, may be cooperative in another region. (Cited in [10].)

Chandra and coworkers have shown how this constraint manifests itself during glycolysis, the process by which cells convert glucose into adenosine triphosphate [33]. Under certain conditions, metabolites involved in glycolysis display oscillations. A large number of models ranging in complexity have been used to recreate these oscillations and aspects such as synchronization. However, the precise reason for these oscillations has not been clear. Chandra *et al.* considered a minimal model of glycolytic oscillations that focused on allosteric activation of phosphofructokinase (PFK) by adenosine monophosphate (AMP) and used this model to demonstrate that, as the control gain increases, the steady-state response decreases, but this is accompanied by an amplification in the gain at mid-frequencies. For sufficiently high feedback gain, this increase can even manifest itself as sustained oscillations. Thus, glycolytic oscillations arise as the inevitable tradeoff of desiring low sensitivity at low frequencies and which can be quantified by Bode's integral.

## 3. Extracting Information in the Presence of Noise

The proper function of cells and organisms depends on their ability to sense their environment and to make correct decisions based on these observations. Both these steps are hampered by stochastic fluctuations inherent in biochemical systems, which have their origin in the random collisions that take place at the molecular level. These fluctuations are usually referred to as *noise*, as they tend to mask the mean level, or signal. Engineering systems are no different, and complex and elaborate theory has evolved for studying and handling this stochasticity. These tools have found their way into the study of biological signaling pathways, and are increasingly making an impact. Here we do not go into the details of how the fluctuations arise or how they are best incorporated into mathematical models, as there are excellent reviews on both these subjects [34,35]. Rather, we focus on how engineering principles arise in studying these systems.

### 3.1. Statistical Inference and Bayes's Rule

To make well-informed decisions, cells must be able to sense their environments accurately. This process is usually done with cell-surface receptors in a reversible binding process we describe by

**Figure 4.**
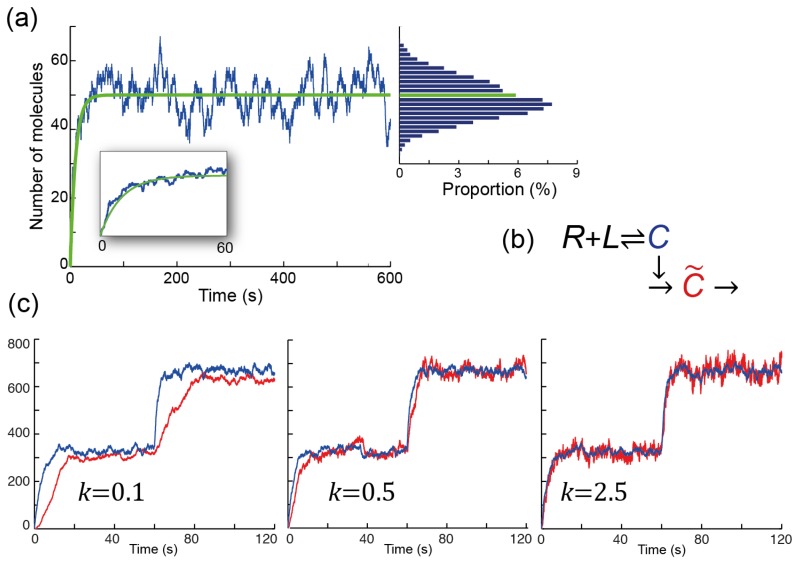
**Coping with stochasticity in signaling. (a)** Stochastic simulation of the binding reaction *R* + *L* ⇌ *C*. The simulation assumes that there are 100 receptors, all unbound initially. Ligand is applied at time zero at a concentration for which the binding and unbinding rates are equal to each other (*k*_off_ = *k*_on_*L* = 0.2 s^−1^). The green line shows the expected number of occupied receptors over time, which is given by the solution to the deterministic ordinary differential Equation 4. The blue line shows how the number of occupied receptors fluctuates over time. The inset focuses on the first 60 seconds. The bar graph shows the distribution for the number of occupied receptors after 100 s (to eliminate the effect of the initial period). In this simulation there was a slight bias as the highest proportion occurred for a number below the expected value (shown by the green graph); **(b)** Scheme for filtering fluctuations; **(c)** Three stochastic simulations of the scheme in panel **(b)**. The binding reactions are as in panel (**a**), but the total number of receptors is 1000. The ligand levels are such that *k*_on_*L* = 0.1 s^−1^ for the first 60 seconds, and *k*_on_*L* = 0.4 s^−1^ thereafter. Note how the lower bandwidth (smaller *k*) leads to smaller deviations, but a lag before the estimate (*C̃*, shown in red) reaches the actual value (*C*, shown in blue). High bandwidths (larger *k*) eliminate the lag, but amplify the noise: the variance in the red signal is larger than that of the blue signal. Intermediate values trade off between the noise and the lag.

R+L⇌C

where the receptor (*R*) binds to a molecule of the ligand (*L*) and becomes occupied (*C*). The deterministic differential equation

(4) $$\frac{\text{d}C(t)}{\text{d}t}=-k_{\text{off}}C(t)+k_{\text{on}}L(R_{\text{T}}-C(t))$$

recreates changes in the expected number of occupied receptors but does not account for fluctuations (Figure 4(a)). If a cell is to make an accurate decision based on receptor occupancy, it must do so not based on the actual external signal (we assume that the concentration of the ligand captures this accurately, though even in this case, there will be fluctuations that can mask its true nature [36]), but on the sensed signal, which is receptor occupancy In mathematics and statistics, this is usually the realm of *statistical inference* [37]. In this field, we make a distinction between the observed or measured signal (which in our case is the steady-state receptor occupancy, *C*) and the environmental signal *L*. We seek to infer the state of the environment given measurements of receptor occupancy. Because of stochasticity, there is no single measure for either; instead, we work in a probabilistic sense and ask, what is the conditional probability that the system is in state (or has concentration) *L* if level *C* is being observed? The optimal solution to this problem is based on Bayes's rule:

$$P(L|C)=\frac{P(C|L)}{P(C)}P(L)$$

Note that, in our example, receptor occupancy is related to the conditional probability *P*(*C*∣*L*), which specifies the likelihood that for a given ligand concentration a certain level of receptor occupancy is obtained. This expression can be rewritten as

$$P(L|C)=\frac{P(C|L)}{\sum_{i}P(C|L_{i})P(L_{i})}P(L)$$

where the summation in the denominator is taken over all possible states of the environment. It is also worth noting that to solve this problem, one must have a probabilistic description of the environment (that is, *P*(*L_i_*)) as well as a suitable model of how these environmental signals give rise to the observed signal (*P*(*C*∣*L_i_*)).

As an example of this optimal statistical inference, Libby *et al.* considered a simple model of the lac operon in *E. coli* [38]. The lac operon is responsible for up-regulating enzymes needed to metabolize lactose. These enzymes are not found constitutively in cells, but are only produced when lactose is present and glucose is absent in the environment. Libby *et al.* assumed that the environment can be described by two states, corresponding to either an environment that is rich in lactose and poor in glucose, in which cells respond by expressing the lac operon, or one that is poor in lactose and rich in glucose, where expression should be repressed. They considered a number of probability distributions for the various terms in the expression for *P*(*L*∣*C*) (note that there are only two terms in the summation) and identified a unique posterior probability distribution that matched the observed lac operon transcription data. In doing so they demonstrated that the lac operon regulatory network solves an optimal statistical inference problem.

### 3.2. Noise Suppression through Temporal Filtering

The inference problem considered above relies on a probabilistic description of the environment and the observations that can be expected. An alternate view of how the non-deterministic nature of the signaling system gives rise to fluctuations is possible. Here we assume that the observed signal, now denoted by *C*(*t*), is a time-varying observation of the environment, which can be represented by a constant ligand concentration, *L*. The expected level of receptor occupancy (assuming that the concentration of the ligand is constant) is

$$C*=\frac{L}{L+K_{D}}R_{\text{T}}$$

where *K_D_* = *k*_off_/*k*_on_ is the dissociation constant for the receptor. Over time, *C*(*t*) fluctuates around C*, but this fluctuation has zero mean (Figure 4(a)). Estimates of C* based on *C*(*t*) will be improved if we average our observations over time, which leads to a low pass filter [39–42]. This low-pass filtering can be carried out by the following reaction scheme

$$Ø\overset{kC}{\to }\tilde{C}\overset{k}{\to }Ø$$

whose expected response can be described by the differential equation:

$$\frac{\text{d}\tilde{C}(t)}{\text{d}t}=-k\left(\tilde{C}(t)-C(t)\right)$$

where *C̃*(*t*) denotes the estimate of *C*(*t*) and the parameter *k* specifies the filter's *bandwidth*—this is the highest frequency for which *C*(*iω*) is not attenuated by the filter. The latter can best be observed in the frequency domain by computing the transfer function of the filter:

$$\frac{\tilde{C}(i\omega )}{C(i\omega )}=\frac{k}{k+i\omega }$$

This transfer function's gain is $k/\sqrt{k^{2}+\omega^{2}}$. For frequencies smaller than *k*, the gain is approximately one. However, if *ω* ≫ *k*, then the gain decreases as *k*/*ω*. Thus, lower bandwidths (smaller *k*) filter out more noise, giving rise to better estimates as measured by the variance of the distribution (Figure 4(c)). However, this improved estimate is obtained by including a longer period of observation, and this can cause a significant delay before changes in the environment are detected.

As we see that the bandwidth has an important effect on the sensed signal, it is worth asking whether there is an optimal bandwidth. A number of reports have considered this question with respect to the signaling pathway controlling chemotaxis in *E. coli* [39,41,43,44]. As mentioned above, a step change in chemoattractant gives rise to a transient response that is followed by perfect adaptation to the pre-stimulus level—a process that can be described as temporal differentiation. In the wild, bacteria are not likely to experience step changes in chemoattractant concentrations, but instead experience gradients of chemoattractant concentration. At least to first degree, these can be approximated as temporal ramps in receptor occupancy that, when differentiated, give rise to a signal that is proportional to the steepness in the gradient and that cells can use to guide their motion. However, binding noise, which is amplified by the temporal differentiation process, perturbs this signal. Thus, cells must filter out the effect of noise to make well-informed decisions. This is done, by low-pass filtering. However, because of biophysical limitations imposed by bacterial swimming—cells experience rotational diffusion that does not allow them to maintain a straight course for more than a few seconds, there is a limit to how far back into the past the low pass filter can go. Estimates based on biophysical constraints have been made [36,41,45], but Andrews *et al.* used an engineering approach to estimate the optimal low-pass filter. Using a model of receptor binding and incorporating known physical limits, they posed the problem as an optimal estimation problem and used the Kalman filter theory [46] to obtain the optimal filter. A comparison of its transfer function to that observed in cells showed strong agreement [43]. Interestingly, the Kalman filter is a special case of an optimal Bayes inference under the assumptions that the noise is Gaussian (a reasonable assumption for binding) and that the system is linear.

## 4. Information-Theoretic Analyses of Signaling Pathways

To a great extent, cell survival requires the ability to make informed decisions based on imprecise measurements of the external environment. Though filtering can help reduce the effect of random perturbations, even the best filtering may not sufficiently remove noise to allow adequate information about the environment to be transmitted to the cell. Prompted by the problem of transmitting signals reliably, Claude Shannon developed a framework that is now known as *information theory* [47,48]. This theory, which is sufficiently general that can be used to understand signal transduction in cell signaling, is only now beginning to have an impact in this biological setting [49–53].

### 4.1. Quantifying the Amount of Information in a Signal

Before seeing how the theory can be used to gain insight into biological systems, it is useful first to understand how information is quantified. The framework is probabilistic. A random signal *X* is assumed to be in one of a number of states, say *x_i_*, according to a probability density function *p*(*x*). The *entropy* of the signal is given by *H*(*X*) = − Σ *p*(*x_i_*) log_2_ *p*(*x_i_*), and is measured in bits. The entropy is a measure of the uncertainty in the variable. For example, if *X* can be in one of two states (say “high” or “low”) with equal probability, then $p(x_{\text{high}})=p(x_{\text{low}})=\frac{1}{2}$, and the entropy is equal to one bit. If the signal is equally likely to be in one of four states, then the entropy is two bits.

A related notion is that of *mutual information*. For two variables *X* and *Y* the mutual information

$$I(X;Y)=H(X)-H(Y|X)=-\sum_{x,y}p(x_{i},y_{j})\log_{2}\frac{p(x_{i},y_{j})}{p(x_{j})p(y_{i})}$$

is the reduction in uncertainty in one variable assuming that the other is known; it is symmetric (*I*(*Y; X*) = *I*(*X; Y*)), so that we can talk about the mutual information between two variables.

**Figure 5.**
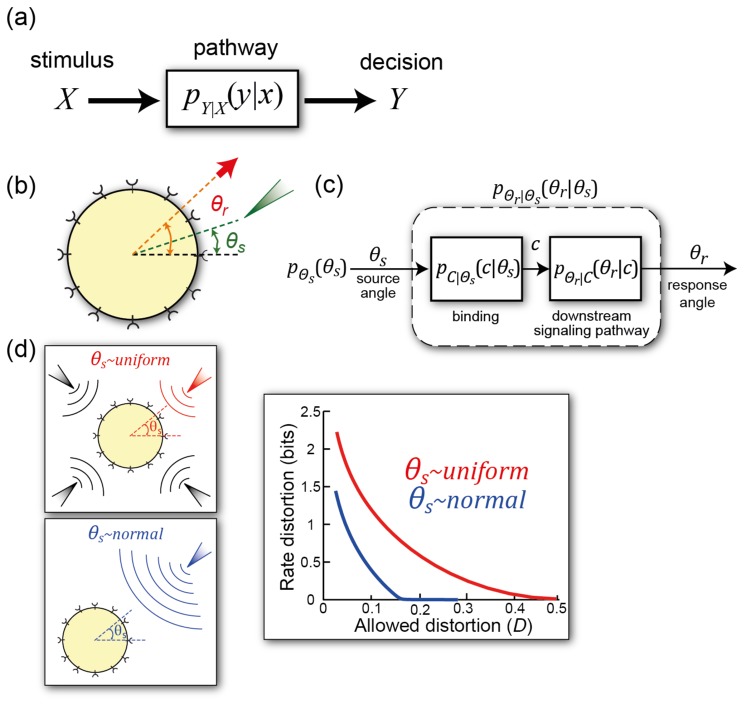
**Information-theoretic approach to cell signaling. (a)** General scheme for dealing with information transmission. The stimulus signal *X* gives rise to a decision *Y*. The signaling pathway describes the channel over which the information flows, and this is characterized by the conditional probability *p_Y_*_∣_*_X_*(*y*∣*x*); **(b)** Gradient sensing in an information-theoretic framework. The external signal *θ_s_* describes the direction of the chemoattractant gradient. The response *θ_r_* is the angle at which the cell moves (or can also describe the spatial response of intracellular markers, such as the concentration of PIP_3_ in response to the gradient [54]); **(c)** Details of the signaling system separating binding and downstream signals; **(d)** Assumptions placed on the external signal. It can be assumed to be uniformly distributed in space or showing some preferential direction. The different assumptions lead to different rate distortion functions. The distortion function is $\frac{1}{2}(1-\cos(\theta_{r}-\theta_{s}))$. These curves display the minimum amount of information needed to ensure that the distortion does not exceed *D*. Any real system must operate at a point above them. Panels (**b**)–(**d**) are used with permission under the Creative Commons Attribution license from [55].

One of Shannon's remarkable results was to provide an upper bound on the information transmission capability of a communication channel. The set-up is as in Figure 5(a). Here, the signal *X* is the signal to be transmitted, and *Y* is the received signal. The nature of the channel dictates the joint probability density function between *X* and *Y*. For example, if data transmission is over a channel that introduces additive white Gaussian noise (AWGN), then for any input *x* ∈ *X*, the received signal is *y* = *x* + *n*, where *n* is a zero-mean Gaussian random variable with variance *σ*^2^. Shannon proved that the *channel capacity*—the maximum rate at which information can be transmitted without any loss in fidelity—is given by the greatest mutual information between *X* and *Y*, where the maximum is computed over all possible input probability density functions. In the case of the AWGN channel, this is infinite unless further constraints are placed on the signal *X*. Typically, this is in the form of a finite power: $P=\frac{1}{T}\int_{0}^{T}{\left|x(t)\right|}^{2}\text{d}t$. In this case, the channel capacity can be shown to be $C=\frac{1}{2}\log_{2}(1+\frac{P}{\sigma^{2}})$, and this is achieved when the input signal is also a zero-mean Gaussian with variance *P*.

Cheong *et al.* sought to measure the mutual information in a biological signaling network [56]. They stimulated mouse fibroblasts with tumor necrosis factor (TNF) at various concentrations and measured the concentrations of the transcription factor NF-*κ*B. They found that the system could only produce slightly less than one bit; that is, it can only really discriminate between the presence and absence of TNF. Their results also suggest that the relatively low information processing capabilities of the system can be attributed to an information bottleneck around the receptor.

This study also showed that negative feedback has a limited role in improving the information capability of the network. Lestas *et al.* considered this question [57] for the problem of reducing stochastic fluctuations in the number of molecules of a system involving feedback control. The system involves two chemical species. Molecule *X* controls the rate of production of molecule *Y*. The latter provides feedback, controlling the rate of production of *X*. The precise form of this control, denoted *U*, is not specified. Instead, arbitrarily complex signaling systems are considered with the only restriction being that the system be *causal*—that is, the control signal can only use past values of *Y*. Information-theoretic methods provided lower bounds on the standard deviation in the number of molecules. These bounds decrease only with the quartic root of the number of signaling events, showing that increasing accuracy is prohibitively expensive.

### 4.2. Information Transmission in Binary Decision Processes

The measurements of the NF-*κ*B system suggest that the system is processing binary information (the presence or absence of TNF) to make a binary decision (whether to initiate transcription or not). Binary decisions such as this one are quite common in biology and include the lac operon system described above. A branch of information theory known as *rate distortion theory* has been used to study the effectiveness of this class of decisions. Unlike Shannon's channel capacity theory, which deals with lossless information transmission, rate distortion theory assumes that some error (the distortion) is allowed. The amount of distortion is measured using the rate distortion function. In a binary system, one possible distortion function would be the Hamming distance [58], which is one if the wrong decision is made and zero otherwise. For example, in the lac operon, if lactose is present and the operon is not activated, then the distortion equals one. Rate distortion theory allows one to pose questions such as: what stimulus–response map (expressed as the conditional probability *p*(*Y*∣*X*)) achieves a pre-specified level of performance (expressed as a distortion level) while minimizing the required transmission cost (the number of bits of mutual information between *X* and *Y*)? Using this framework, it is possible to show that a number of responses observed in biological signaling pathways arise as the optimal response of systems [59]. For example, by replacing the Hamming distance with a distortion function that penalizes incorrect decisions gradually, depending on where the stimulus concentration is in relation to a threshold, the optimal stimulus–response map is sigmoidal [59]. Moreover, requiring higher fidelity (lower allowed distortion) leads to steeper sigmoidal functions as measured by the Hill coefficient. In this framework, responses that include hysteresis or irreversibility appear as optimal strategies.

### 4.3. Information Processing During Eukaryotic Chemotaxis

Not all the relevant information in cell signaling is binary. For example, chemotactic cells must interpret spatial differences (as small as 1%) in the concentration of diffusible chemical in order to guide their locomotion. The information-processing capabilities of these cells has led to a number of interesting studies in both amoebae [55,60,61] and axons [62,63].

To estimate mutual information between the chemoattractant gradient and the spatial response, the cell membrane can be divided into *n* segments, each with *N*/*n* receptors (*N* is the total number of receptors.) Assuming a simple model of ligand binding (as in Section 3.1), Fuller *et al.* calculated that the mutual information between the direction of the gradient (*θ_s_*) and the concentration of occupied receptors (*C*) is given by

$$I(C;\theta_{s})\approx \frac{NK_{D}\;Lp^{2}}{16{\left(K_{D}+L\right)}^{2}}$$

where *p* is the gradient in chemoattractant concentration, expressed as a percentage of the mid-point of receptor occupancy [60]. These calculations show that the mutual information is a biphasic curve of the concentration, with a peak at *L* = *K_D_*, but that it increases with the gradient steepness. Because this expression represents the mutual information between chemoattractant gradient and receptor occupancy, they termed this the *external* mutual information. They went on to compute the mutual information between receptor occupancy and the cell's directional response, which is the angle of cell motility (*θ_r_*). Using experimentally observed tracks of cells moving in response to a gradient, they measured the direction of response and found that the mutual information between chemoattractant gradient and directional response peaks at about 0.5 bits and is relatively constant for gradients steeper than about 5 %. These results are similar to studies of axonal chemotaxis using a Bayesian model [62,63], which show that for very shallow gradients the response increases with gradient steepness but eventually saturates.

Rate distortion theory has also been used to compute the optimal stimulus response maps for chemotaxis [55]. This optimal system was in strong agreement to that of a model used to explain the gradient sensing pathway of *Dictyostelium* cells (the so-called, local-excitation, global-inhibition model [25]). This study also demonstrated that the presence of prior information—as can be expected when cells are persistently moving in a stable chemoattractant gradient—improves chemotactic efficiency However, if the prior information is in conflict with the information from receptor occupancy (as might be the case if the direction of the gradient changes) then the cell trades off between the two sources of information. These results help to explain why polarized *Dictyostelium* cells perform gradual turns in response to changing gradient directions.

## 5. Conclusions

This review has highlighted some successes in understanding how biological systems can be studied using analysis and design tools from engineering. Our coverage is by no means exhaustive. Among the areas that we have not touched upon include tools developed to study biological reactions when the number of molecules is sufficiently small that continuum descriptions are not possible (see, for example, [64,65]), the development of algorithms for identifying parameters in biological networks (e.g., [66–68]), and the use of computational tools to guide synthetic biology (e.g., [69–71]).

The analogy between biological and engineering systems can sometimes be carried too far, as the general setting between the two is quite different. As an example, we turn to the standard paradigm of control systems that delineates between two subsystems: the *plant*, which is specific *a priori* and cannot be altered, and the *controller*, which is the system that is to be designed. This distinction is not necessarily possible in biological signaling. While we may be able to view certain aspects of biological systems as “controllers” (we do, after all, talk about regulation), most of the time we need to consider the system as a whole. Thus, straightforward application of these design tools may not be possible.

Where they are most suitable is when they highlight fundamental limitations and can thus be used to consider the performance of biological systems in relation to some optimal level of performance, or in light of tradeoffs that need to be made. Interestingly, this *normative* view of biology that specifies what a system “should do” is quite common in studying neural systems [72,73]. It is possible that the complexity of the neural system necessitates that systems be understood in the context of these optimality principles. In contrast, the development of mathematical models of cell signaling from known biochemistry is possible, and it has not been necessary to seek these underlying principles. The studies reviewed here show, however, that cell signaling does operate in this utility-maximizing regime. As the complexity of the signaling systems that we seek to understand increases, we can expect this point of view to gain greater prominence.

## A. Appendix

This appendix provides some more details about the mathematical derivations as well as simulations.

### A.1. Derivation of the Transfer Function

If the input and set-point are denoted by *u*_0_ and (*x*_0_,*y*_0_,*z*_0_), respectively, we compute the partial derivatives in the Jacobian [4] to get

(5) $$\frac{\text{d}}{\text{d}t}\left[\begin{array}{c} \delta x\\ \delta y\\ \delta z \end{array}\right]=\left[\begin{array}{ccc} -\alpha & 0 & 0\\ 0 & -\beta & \epsilon \\ \delta & -k & -\gamma \end{array}\right]\;\left[\begin{array}{c} \delta x\\ \delta y\\ \delta z \end{array}\right]+\left[\begin{array}{c} \theta \\ 0\\ 0 \end{array}\right]\delta u$$

where

$$\begin{array}{ll} \alpha & =\frac{k_{UX}k_{X}u_{0}}{{\left(1-x_{0}+k_{X}\right)}^{2}}+\frac{k_{-X}k_{X}^{\prime }}{{\left(x_{0}+k_{X}^{\prime }\right)}^{2}}\\ \beta & =\frac{k_{ZY}k_{Y}z_{0}}{{\left(1-y_{0}+k_{Y}\right)}^{2}}+\frac{k_{-Y}k_{Y}^{\prime }}{{\left(y_{0}+k_{Y}^{\prime }\right)}^{2}}\\ \gamma & =\frac{k_{XZ}k_{Z}x_{0}}{{\left(1-z_{0}+k_{Z}\right)}^{2}}+\frac{k_{-Z}k_{Z}^{\prime }y_{0}}{{\left(z_{0}+k_{Z}^{\prime }\right)}^{2}}\\ \delta & \begin{array}{lll} =\frac{k_{XZ}(1-z_{0})}{1-z_{0}+k_{Z}}, & \epsilon & =\frac{k_{Z}Y(1-y_{0})}{1-y_{0}+k_{Y}} \end{array}\\ \theta & \begin{array}{lll} =\frac{k_{UX}(1-x_{0})}{1-x_{0}+k_{X}}, & k & =\frac{k_{-Z}k_{Z}^{\prime }z_{0}}{z_{0}+k_{Z}^{\prime }} \end{array} \end{array}$$

Taking the Fourier transform of Equation 5 and collecting terms yield the transfer function

$$\begin{array}{ll} H(i\omega )=\frac{\Delta Z(i\omega )}{\Delta U(i\omega )} & =\left[\begin{array}{ccc} 0 & 0 & 1 \end{array}\right]\;{\left[\begin{array}{ccc} i\omega +\alpha & 0 & 0\\ 0 & i\omega +\beta & -\epsilon \\ -\delta & k & i\omega +\gamma \end{array}\right]}^{-1}\;\left[\begin{array}{c} \theta \\ 0\\ 0 \end{array}\right]\\ & =\frac{\delta (i\omega +\beta )\theta }{\left(i\omega +\alpha )({\left(i\omega \right)}^{2}+\left[\beta +\gamma \right](i\omega )+\beta \gamma +k\epsilon \right)} \end{array}$$

To obtain the sensitivity function, we write *H*(*iω*) as

$$\frac{\delta \theta }{\left(i\omega +\alpha )(i\omega +\gamma \right)}\times \frac{\left(i\omega +\beta )(i\omega +\gamma \right)}{{\left(i\omega \right)}^{2}+[\beta +\gamma ](i\omega )+\beta \gamma +k\epsilon }$$

where the first term is just *H*(*iω*) with the feedback term set to zero (*k* = 0).

If 1 − *y*_0_ ≫ *k_Y_* and $y_{0}\gg k_{Y}^{\prime }$, then *β* ≈ 0, in which case

$$H(i\omega )\approx \frac{(i\omega )\delta \theta }{\left(i\omega +\alpha )({\left(i\omega \right)}^{2}+\gamma (i\omega )k\epsilon \right)}$$

and

$$S(i\omega )\approx \frac{\left(i\omega )(i\omega +\gamma \right)}{{\left(i\omega \right)}^{2}+\gamma (i\omega )+k\epsilon }$$

### A.2. Simulations

The simulations were computed using MATLAB (Mathworks, Natick, MA). The deterministic simulations of Figure 1(c) used the ode23.m solver, using parameters *k_UX_* = *k_XZ_* = 0.2s^−1^, *k*_−_*_X_* = *k*_−_*_Y_* = *k*_−_*_Z_* = 0.2s^−1^, $k_{X}=k_{X}^{\prime }=0.25$, $k_{Y}=k_{Y}^{\prime }=0.0001$, and $k_{Z}=k_{Z}^{\prime }=0.25$.

Stochastic simulations were carried out using Gillespie's stochastic simulation algorithm [74].
